# Essential contribution of CCL3 to alkali-induced corneal neovascularization by regulating vascular endothelial growth factor production by macrophages

**Published:** 2008-09-05

**Authors:** Peirong Lu, Longbiao Li, Yu Wu, Naofumi Mukaida, Xueguang Zhang

**Affiliations:** 1 Clinical Immunology Key Laboratory of Jiangsu Province, the First Affilated Hospital of Soochow University, Suzhou, China; 2 Department of Ophthalmology, the First Affiliated Hospital of Soochow University, Suzhou, China; 3 Biotechnology Institute, the First Affilated Hospital of Soochow University, Suzhou, China; 4 Division of Molecular Bioregulation, Cancer Research Institute, Kanazawa University, Kanazawa, Japan

## Abstract

**Purpose:**

To evaluate the roles of CCL3 and its specific chemokine receptors, CCR1 and CCR5, in alkali-induced corneal neovascularization (CNV).

**Methods:**

Chemical denudation of corneal and limbal epithelium was performed on wild-type (WT) BALB/c mice and CCL3-, CCR1-, and CCR5-deficienct (knockout [KO]) counterparts. Two weeks after injury CNV was quantified by immunostaining with anti-CD31. Angiogenic factor expression and leukocyte accumulation in the early phase after injury were quantified by reverse transcription polymerase chain reaction (RT–PCR) and immunohistochemical analysis, respectively.

**Results:**

Alkali injury augmented the intraocular mRNA expression of CCL3 and its receptors, CCR1 and CCR5, together with a transient infiltration of F4/80 positive macrophages and Gr-1 positive neutrophils. Compared with WT mice, CCL3-KO and CCR5-KO mice but not CCR1-KO mice exhibited reduced CNV two weeks after injury both macroscopically and microscopically as evidenced by CD31 positive areas. Concomitantly, the infiltration of F4/80 positive macrophages but not Gr-1 positive neutrophils was significantly attenuated in CCL3-KO mice compared with WT mice. Intracorneal infiltration of CCR5 expressing cells was significantly impaired in CCL3-KO mice compared with WT mice. Alkali injury induced a massive increase in the intraocular mRNA expression of a potent angiogenic factor, vascular endothelial growth factor (VEGF), in WT mice whereas these increments were severely retarded in CCL3-KO mice. Moreover, CCL3 enhanced VEGF expression by murine peritoneal macrophages at both the mRNA and the protein level. Furthermore, topical CCL3 application restored CNV, which was macroscopically and microscopically reduced in CCL3-KO mice after two weeks to levels similar to those found in WT mice.

**Conclusions:**

In alkali-induced CNV, CCL3 induced macrophages to infiltrate and produce VEGF by binding to CCR5 but not to CCR1 and eventually promoted angiogenesis.

## Introduction

The cornea is characterized by an absence of blood vessels under physiologic conditions [1]. Corneal avascularity is maintained by a balance between angiogenic and anti-angiogenic molecules [2-6] and is required for optical clarity and the maintenance of vision. Thus, corneal neovascularization (CNV) can lead to impaired vision when it arises from any cause including corneal infections, misuse of contact lenses, chemical burns, and inflammation [7-9]. In most of these conditions, a large number of neutrophils infiltrate into the cornea before the onset of CNV followed by an infiltration of monocytes/macrophages. Although neutrophils are presumed to be involved in CNV, we have previously shown that alkali-induced CNV developed independently of granulocyte infiltration [10].

Leukocyte infiltration is regulated by coordinative actions of adhesion molecules and chemokines with the chemokine receptor expression pattern on leukocytes determining their responsiveness to a given chemokine [11]. Monocytes/macrophages express a distinct set of chemokine receptors including CCR1, CCR2, CCR5, and CX3CR1 on their cell surface [12-14]. We have previously found a potent angiogenic factor, vascular endothelial growth factor (VEGF), which was detected in intraocularly infiltrating monocytes/macrophages before CNV development [10]. CNV could be consistently attenuated by genetic ablation of either the *CCR2* or *CCR5* gene [15,16], which also reduced intraocular VEGF production. In contrast, several other groups have provided evidence to indicate that infiltrating macrophages have anti-angiogenic activities in choroidal neovascularization [17]. In line with this notion, we also observed that intraocularly infiltrated CX3CR1-positive macrophages expressed anti-angiogenic molecules such as thrombospondins and were protective against alkali-induced CNV [18]. Thus, the monocyte/macrophage population may be heterogeneous in terms of angiogenic activities, which depends on their chemokine receptor expression pattern.

We previously observed that CCR1 was expressed in endothelial cells in human hepatoma tissue [19]. Furthermore, both CCR1-knockout (KO) and CCL3-KO mice exhibited impairment in carcinogen-induced hepatocarcinogenesis with reduced macrophage infiltration and intra-tumor neovascularization [20]. These observations would imply that involvement of the CCL3-CCR1 axis in neovascularization is essential. Because CCL3 can also bind to CCR5 as well as CCR1 [21], we compared the molecular pathological changes between WT mice and mice that were deficient in CCL3, CCR1, or CCR5 in a frequently used ocular neovascularization model, alkali-induced CNV [10,15,16,18], to address the roles of CCL3 and its receptors in CNV. We provided definitive evidence to indicate involvement of the CCL3/CCR5 axis but not the CCL3/CCR1 axis in alkali-induced CNV.

## Methods

### Reagents and antibodies

Recombinant CCL3/MIP-1α (270-LD) and goat anti-mouse CCL3 antibodies were obtained from R&D Systems (Minneapolis, MN). Rat anti-mouse F4/80 (clone A3–1) monoclonal antibody (mAb) was from Serotec (Oxford, UK). Polyclonal rabbit antibody to CD31 (ab28364) was purchased from Abcam (Cambridge, UK). Rat anti-mouse CD31 (MEC13.3), purified rat anti-mouse-Ly-6G and Ly-6C (Gr-1) mAbs (clone RB6–8C5), and purified rat anti-mouse CCR5 mAb (clone C34–3448) were purchased from BD PharMingen (San Diego, CA). Goat anti-CCR1 pAb (C-20) was obtained from Santa Cruz Biotechnology (Santa Cruz, CA). Alexa Fluor (AF) 488 donkey anti-rat IgG (H^+^L), AF594 donkey anti-rabbit IgG (H^+^L), and AF594 donkey anti-goat IgG (H^+^L) were purchased from Invitrogen (Shanghai, China).

### Mice

CCL3-deficient (CCL3-KO) mice were obtained from Jackson Laboratories (Bar Harbor, ME). CCR1-deficient (CCR1-KO) and CCR5-deficient (CCR5-KO) mice were generous gifts from Dr. P. M. Murphy and Dr. J. L. Gao (NIADID, NIH, Bethesda, MD) [22] and from Dr. Kouji Matsushima (University of Tokyo, Tokyo, Japan), respectively [23]. These deficient mice were backcrossed with BALB/c for more than eight generations. Pathogen-free BALB/c mice were obtained from Clea Japan (Yokohama, Japan) and were designated as WT mice. All animal experiments were performed under specific pathogen-free conditions in the Institute for Experimental Animals (Kanazawa University, Kanazawa, Japan) in accordance with the ARVO Statement for the Use of Animals in Ophthalmic and Vision Research and complied with the standards set out in the Guidelines for the Care and Use of Laboratory Animals of Kanazawa University.

### Alkali-induced corneal injury model

Corneal injury was induced by placing a 2 mm filter disc saturated with 1 N NaOH onto the left eye of the mouse as previously described [10,18]. In some experiments, the alkali-treated eyes received 5 μl of CCL3 preparation dissolved in 0.2% sodium hyaluronate (Sigma-Aldrich, St. Louis, MO) at a concentration of 3 μg/ml or 5 μl of 0.2% sodium hyaluronate as the vehicle twice a day for seven days immediately after the alkali injury. At the indicated time intervals, mice were killed, and whole eyes were removed. The eyes were fixed in 10% neutrally buffered formalin or were snap frozen in optimal cutting temperature (OCT) compound (Sakura Finetek, Torrance, CA). In some mice, the corneas were removed from both eyes, placed immediately into RNALate (Qiagen, Tokyo, Japan), and kept at −86 °C until total RNA extraction was performed. Each experiment was repeated at least three times.

### Biomicroscopic examination

Eyes were examined under a surgical microsystem (Lecia MZ16; Leica Microsystems GmbH, Wetzlar, Germany) 14 days after alkali injury by two independent observers with no prior knowledge of the experimental procedures as described previously [10,18].

### Histological and immunohistochemical analysis

The paraffin-embedded tissues were cut into 5 µm thick slices and were then subjected to hematoxylin and eosin staining. Other sections were deparaffinized with xylene and rehydrated through graded concentrations of ethanol for immunohistochemical detection of F4/80 positive, CCL3 positive, CCR1 positive, or CCR5 positive cells as described previously [20]. The numbers of F4/80 positive cells were counted at 200 fold magnification in five randomly chosen fields of corneal sections from each animal [18,20] by an examiner with no prior knowledge of the experimental procedures. The numbers of positive cells/mm^2^ were calculated.

### A double-color immunofluorescence analysis

A double-color immunofluorescence analysis was performed to determine the cells expressing CCR5 and CCR2. Briefly, the fixed cryosections (8 µm thick) were incubated with PBS containing 1% normal donkey serum and 1% BSA to reduce nonspecific reactions. Thereafter, the sections were incubated with a combination of rat anti-CCR5 and rabbit anti-CCR2 overnight at 4 °C. After being rinsed with PBS, the sections were then incubated with a combination of Alexa Fluor 488 donkey anti-rat IgG and Alexa Fluor 594 donkey anti-rabbit IgG (1/100) for 40 min at room temperature in the dark. Finally, the sections were washed with PBS, and immunofluorescence was visualized in dual-channel mode with a fluorescence microscope (Olympus, Tokyo, Japan). Images were processed using Adobe Photoshop software version 7.0 (Adobe Systems, Tokyo, Japan).

### Enumeration of corneal neovascularization

Deparaffinized sections (5 µm thick) and fixed cryosections (8 µm thick) were stained using rabbit anti-CD31 antibody (ab28364) [10] and rat anti-CD31 antibody (MEC13.3) [18], respectively. The numbers and sizes of the CNV were determined as previously described [10] by an examiner with no prior knowledge of the experimental procedures. Most sections were taken from the central region of the cornea. The numbers and areas of corneal neovascularization were evaluated on at least two sections from each eye.

### Semi-quantitative reverse transcription polymerase chain reaction

Total RNAs were extracted from the corneas or cultured macrophages with the use of RNeasy Mini Kit (Qiagen, Tokyo, Japan), and the resultant RNA preparations were further treated with RNase-free DNase I (Life Technologies Inc., Gaithersburg, MD) to remove genomic DNA. Total RNA (2 μg) were reverse-transcribed at 42 °C for 1 h in 20 μl of reaction mixture containing mouse Moloney leukemia virus reverse transcriptase and hexanucleotide random primers (Qiagen). cDNA (twofold serially diluted) was amplified for *β-actin* (Table 1) to estimate the amount of transcribed cDNA. Then, equal amounts of cDNA products were amplified for the target genes using the primers under the following conditions: denaturation at 94 °C for 2 min followed by the optimal cycles of 30 s at 94 °C, 45 s at 53–57 °C, 1 min at 72 °C, and a final 10 min extension step at 72 °C (Table 1). The amplified polymerase chain reaction (PCR) products were fractionated on a 1.0% agarose gel and visualized by ethidium bromide staining. The band intensities were measured, and their ratios to β-actin were determined with the aid of NIH Image Analysis software.

**Table 1:** Sequences of the primers used for reverse transcription polymerase chain reaction.

**Gene name**	**Sequence**	**Product size (bp)**	**Annealing temperature (°C)**	**PCR cycles**
*CCR1*	F: TTTTAAGGCCCAGTGGGAGTT	475	57	37
	R: TGGTATAGCCACATGCCTTT			
*CCR5*	F:GTCCTCCTCCTGACCACCTT	122	55	38
	R: GGGTTTAGGCAGCAGTGTGT			
*CCL3/MIP-α*	F: ATCATGAAGGTCTCCACCAC	284	56	37
	R: TCTCAGGCATTCAGTTCCAG			
*VEGF*	F: CTGCTGTACCTCCACCATGCCAAGT	509	57	37
	R: CTGCAAGTACGTTCGTTTAACTCA			
*bFGF*	F: CTTCCCACCAGGCCACTT	370	53	38
	R: CTGTCCAGGTCCCGTTTT			
*TSP-1*	F: ACCAAAGCCTGCAAGAAAGA	311	57	37
	R: ATGCCATTTCCACTGTAGCC			
*β-actin*	F: TGTGATGGTGGGAATGGGTCAG	514	55	25
	R: TTTGATGTCACGCACGATTTCC			

All primers used were purchased from Genset Oligos (Kyoto, Japan). The amplification was performed using a GeneAmp^®^ PCR System 9700 (Perkin-Elmer, Foster City, CA). In the table, "F" indicates forward primer and "R" indicates reverse primer.

### Murine peritoneal macrophages isolation and culture

Peritoneal macrophages were obtained as described previously [18]. The cells were suspended in antibiotic-free RPMI medium containing 10% fetal bovine serum (FBS) and incubated in a humidified incubator at 37 °C in 5% CO_2_ in 24 well cell culture plates (Nalge Nunc International Corp., Naperville, IL). Two hours later, non-adherent cells were removed, and the medium was replaced. The cells were then stimulated with the indicated concentrations of murine CCL3 for 12 h. Total RNAs were extracted from the cultured cells and subjected to reverse transcription polymerase chain reaction (RT–PCR) as described above. In another series of experiments, murine macrophages were seeded onto 12 well plates at 5×10^5^ cells/well. After adhesion, the cells were stimulated with the indicated concentrations of murine CCL3 for 24 h in a 37 °C incubator with 5% CO_2_. Supernatants were collected to determine VEGF concentrations using a mouse VEGF ELISA Kit (R&D Systems) according to the manufacturer’s instructions.

### Statistical analysis

The means and the standard error of the mean (SEM) were calculated for all parameters determined in the study. Data were analyzed statistically using one-way ANOVA or two-tailed Student’s *t*-test. A value of p<0.05 was accepted as statistically significant.

## Results

### Intracorneal expression of CCL3 and its receptor, CCR1 and CCR5, after alkali-induced corneal injury

**Figure 1:**
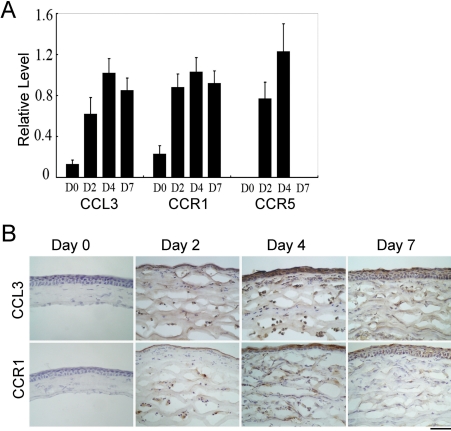
The expression of CCL3 and its receptors in cornea after alkali injury. **A**: Semi-quantitative RT–PCR was performed to assess mRNA expression of *CCL3* and its receptors, *CCR1* and *CCR5*, and the ratios of target gene expression to *β-actin* were determined. All values represent the mean±SEM of three to five independent measurements. **B**: Whole eyes were obtained at 0, 2, 4, and 7 days after alkali injury and processed for immunohistochemical analysis using anti-CCL3 (upper panels) or anti-CCR1 antibodies (lower panels). Representative results from five individual animals are shown. Original magnifications, 400X. Scale bar, 50 μm.

We first examined the expression of CCL3, a ligand for CCR5 and CCR1, in corneas after alkali-induced injury. *CCL3* mRNA was barely detectable in untreated eyes but was markedly increased after alkali injury (Figure 1A). Concomitantly, CCL3 protein was immunohistochemically detected in epithelial cells and infiltrating cells after alkali injury but not in untreated eyes (Figure 1B). Moreover, alkali injury markedly augmented the mRNA expression of specific receptors for *CCL3*, *CCR1*, and *CCR5* (Figure 1A). Furthermore, immunohistochemical analysis demonstrated the infiltration of CCR1 expressing leukocytes, which started two days after the injury and increased thereafter (Figure 1B). These observations suggest that alkali injury induced intracorneal production of CCL3, which in turn attracted CCR1 expressing or CCR5 expressing leukocytes into the cornea.

### Impaired alkali-induced corneal neovascularization in CCL3-KO and CCR5-KO but not CCR1-KO, mice

**Figure 2:**
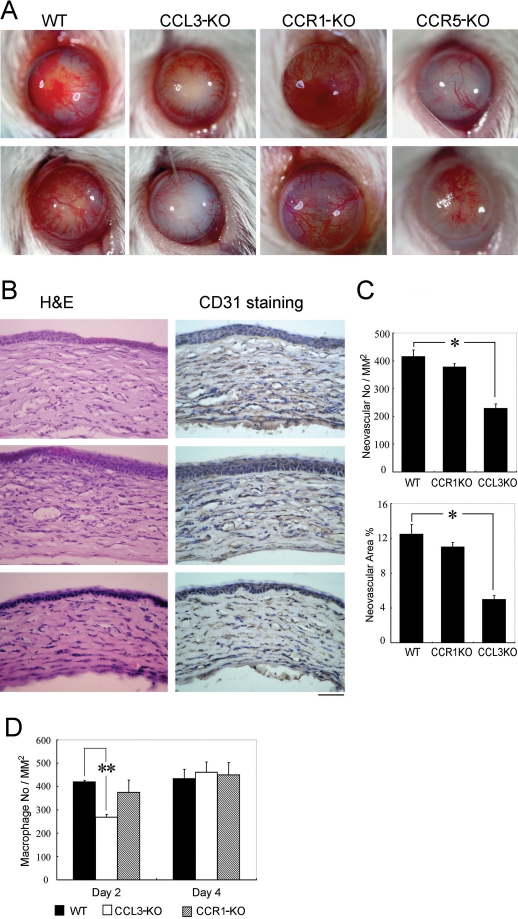
Alkali-induced corneal neovascularization and macrophage infiltration. **A**: The macroscopic appearances of WT, CCL3-KO, CCR1-KO, and CCR5-KO mouse eyes two weeks after alkali injury are illustrated. Representative results from at least 10 animals in each group are shown here. **B**: Corneal tissues were obtained from WT, CCR1-KO, and CCL3-KO mice two weeks after the injury. Tissues were stained with hematoxylin and eosin (left panels) or immunostained with anti-CD31 antibodies (right panels), and representative results from five individual animals are shown. Original magnifications, 400X. Scale bar, 50 μm. **C**: CNV numbers per mm^2^ in hot spots (upper panel) and % CNV areas in hot spots (lower panel) were determined on corneas obtained from WT or KO mice two weeks after the injury. Each value represents the mean and SEM (n=5 animals). An asterisk represents a p<0.05 and that the value was obtained comparing WT and CCL3-KO mice. **D**: The numbers of infiltrated F4/80 positive macrophages were determined two and four days after the injury. Each value represents the mean and SEM (n=5). The double asterisk indicates a p<0.01 and that the value was obtained comparing WT and CCL3-KO mice.

We next explored the effects of genetic ablation of *CCL3*, *CCR1*, and *CCR5* on alkali-induced CNV. CNV was macroscopically evident in WT mice two weeks after the injury as we have previously reported [10]. In line with the previous report [16], macroscopic CNV was markedly attenuated in CCR5-KO mice (Figure 2A). Moreover, macroscopic CNV was markedly reduced in CCL3-KO mice but in not CCR1-KO mice (Figure 2A,B,C). Although corneas are physiologically avascular, alkali injury markedly increased the vascular areas in corneas of WT and CCR1-KO mice to similar extents, but the increment was significantly reduced in CCL3-KO mice (Figure 2B,C). These observations would indicate that the CCL3-CCR5 axis was indispensable for alkali-induced CNV, but the CCL3-CCR1 axis was not.

### Reduced intraocular macrophage infiltration in CCL3-KO but not CCR1-KO mice

We previously observed that Gr-1 positive granulocytes and F4/80 positive macrophages infiltrated injured corneas, reaching their peak levels two to four days after the injury in WT mice [10,18]. Leukocytes, particularly monocytes/macrophages, can be a rich source of angiogenic factors [24-28]. Given the fact that CCL3 recruit macrophages, which express CCR1 and CCR5, we examined the effects of CCL3 deficiency on leukocyte infiltration into the wounded cornea. Neither F4/80 positive macrophages nor Gr-1 positive granulocytes were present in untreated corneas of WT and CCL3-KO mice. Gr-1 positive granulocytes infiltrated to similar extents into the corneas of both WT and CCL3-KO mice after the injury (data not shown). In contrast, F4/80 positive macrophage infiltration was markedly reduced in CCL3-KO mice but not in CCR1-KO mice when compared with WT mice (Figure 2D). Thus, CCL3 may regulate intraocular infiltration of F4/80 positive macrophages but not Gr-1 positive granulocytes.

### Reduced vascular endothelial growth factor expression in CCL3-KO mice after alkali injury

**Figure 3:**
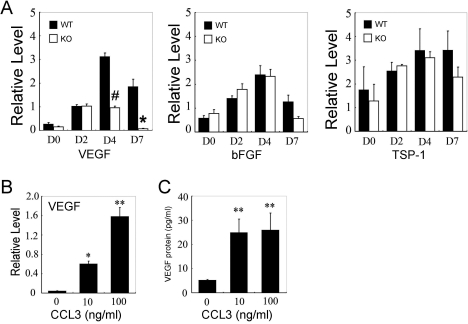
Angiogenic factor expression. **A**: RT–PCR analysis of pro-angiogenic and anti-angiogenic gene expressions in the injured corneas of WT and CCL3-KO mice. RT–PCR analysis was performed on total RNAs extracted from eyes 0, 2, 4, and 7 days after alkali injury, and then the ratios of VEGF to β-actin, bFGF to β-actin, and TSP-1 to β-actin of WT (black bars) and CCL3-KO mice (open bars) were determined. All values represent the mean and SEM (n=3-5 animals). The asterisk denotes a p<0.05; the hash mark denotes a p<0.01 and that the value was obtained comparing WT and KO mice. The effects of CCL3 on VEGF expression by murine peritoneal macrophages is shown in **B** and **C**. **B**: RT–PCR was performed on macrophages incubated with the indicated concentrations of CCL3 for 12 h, and the ratio of VEGF to β-actin was calculated. Each value represents the mean and SEM (n=3). **C**: Murine macrophages were stimulated with either 0, 10, or 100 ng/ml of CCL3 for 24 h. VEGF concentrations in the supernatants were determined with ELISA as described in Methods. The representative results from three independent experiments are shown. The asterisk denotes a p<0.05 and the double asterisk denotes a p<0.01 when compared to untreated.

The balance between angiogenic and anti-angiogenic factors can determine the outcome of angiogenetic processes in various situations. Hence, we examined the mRNA expression of angiogenic and anti-angiogenic factors in corneas after the injury. Alkali injury increased intraocular mRNA expression of an angiogenic factor, basic fibroblast growth factor (*bFGF*), and an anti-angiogenic molecule, thrombospondin (*TSP-1*), in WT and CCL3-KO mice to similar extents (Figure 3A). In contrast, mRNA expression of another potent angiogenic factor, *VEGF*, was markedly augmented in WT mice, and the increase was markedly attenuated in CCL3-KO mice (Figure 3A).

### Enhanced vascular endothelial growth factor expression by murine peritoneal macrophages with CCL3 stimulation

We next examined the effects of exogenous CCL3 on *VEGF* expression by mouse peritoneal macrophages at the mRNA and protein levels. CCL3 markedly enhanced the mRNA expression of *VEGF* by peritoneal macrophages (Figure 3B). Concomitantly, CCL3 increased VEGF protein production by macrophages in a dose-dependent manner (Figure 3C). These observations would indicate that CCL3 can activate macrophages to produce an angiogenic factor, VEGF.

### Simultaneous CCR2 expression by intracorneally infiltrating CCR5 expressing cells

**Figure 4:**
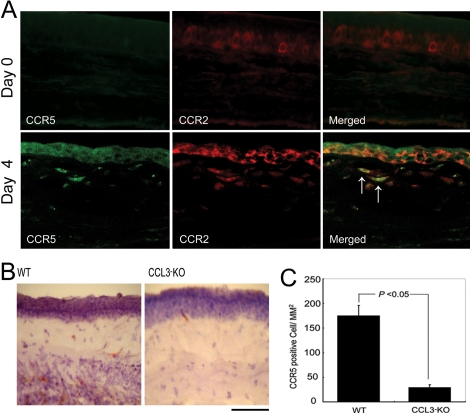
Intracorneal CCR5 positive cell infiltration. **A**: A double-color immunofluorescence analysis of CCR5-expressing cells is illustrated. Corneas were obtained from WT mice 0 and 4 days after the injury. The samples were immunostained with a combination of anti-CCR5 and anti-CCR2 antibodies as described in Methods and observed with fluorescence microscopy (original magnification, 400X). Signals were digitally merged in the right panels. Arrows indicate the double, positively stained cells. Representative results from three independent experiments are shown. **B**: Corneal tissues from WT mice (left panel) or CCL3-KO mice (right panel) obtained four days after the injury were stained with anti-CCR5 Ab. Scale bar, 100 μm. **C**: The numbers of intracorneal CCR5 positive cells four days after the injury were determined as described in Methods, and the mean and SEM are shown here (n=5).

We previously revealed that the CCL2/CCR2 interactions could induce VEGF expression [18]. Hence, we next examined whether intraocularly infiltrating CCR5 expressing cells also expressed CCR2. A double-color immunofluorescence analysis demonstrated that CCR5 expressing cells also expressed CCR2 (Figure 4A). Moreover, alkali injury-induced increases in intracorneal CCR5 positive cell numbers were attenuated in CCL3-KO mice (Figures 4B,C). Thus, it is likely that CCL3 can regulate intraocular infiltration of CCR5 expressing macrophages, which can express VEGF by the CCL2/CCR interactions.

### Restoration of alkali-induced corneal neovascularization in CCL3-KO mice by topical CCL3 application

**Figure 5:**
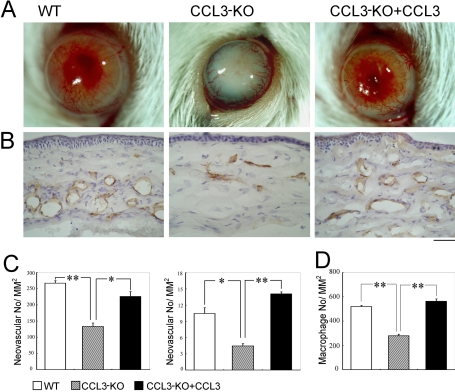
The effects of topical CCL3 application on corneal neovascularization. **A**: Macroscopic appearances of WT, CCL3-KO mice, and CCL3-KO mice topically applied with CCL3 two weeks after alkali injury are shown. Representative results from five animals from each group are shown here. **B**: Corneal tissues were obtained two weeks after the injury from WT, CCL3-KO, and CCL3-KO mice topically applied with CCL3 and were immunostained with anti-CD31 antibodies. Representative results from five individual mice from each group are shown. Original magnification, 400X. Scale bar, 50 μm. **C**: The CNV numbers per mm^2^ in hot spots (left panel) and % CNV areas in hot spots (right panel) were determined. Each value represents the mean and SEM (n=5 animals). **D**: The number of infiltrated F4/80 positive macrophages was determined on WT, CCL3-KO, and CCL3-KO, which were all treated with CCL3, two days after the injury. Each value mean represents both the mean and SEM (n=5). The asterisk denotes a p<0.05, and the double asterisk means a p<0.01 when compared with CCL3-KO (this applies to both **C** and **D**).

Finally, we examined the effects of topical CCL3 application on alkali-induced CNV of CCL3-KO mice. CCL3-KO mice exhibited reduced alkali-induced CNV at both macroscopic and microscopic levels compared with WT mice (Figure 5A-C). Topical CCL3 application restored CNV to an extent similar to that seen in WT mice (Figure 5A-C). Concomitantly, CCL3 treatment reversed the macrophage infiltration in CCL3-KO mice to similar levels as WT mice (Figure 5D). Thus, CCL3 may induce the infiltration of macrophages, which in turn may produce a potent angiogenic factor, VEGF, and eventually promote alkali-induced CNV.

## Discussion

Tissue injury induced the expression of various growth factors, cytokines, and chemokines, which all contribute to tissue repair in a coordinated manner [29]. In cooperation with adhesion molecules, chemokines can regulate the trafficking of various types of leukocytes, which in turn regulate two processes of tissue repair, granulation tissue formation, and neovascularization by producing various growth factors and cytokines [29-32]. Moreover, several chemokines can directly regulate neovascularization [33]. Alkali injury induced a transient macrophage infiltration into eyes with enhanced intraocular CCL3 expression. Several independent groups reported that CCL3 has direct effects on endothelial cells [33] and that its receptor, CCR1, was expressed in certain types of endothelial cells [19,20]. However, CCL3 can restore CNV in CCL3-KO mice to similar levels shown in WT mice. This restoration of CNV is seen even if CCL3 was administered only in the early phase after the injury at the time when the endothelial cells are absent. Thus, CCL3 may not directly target endothelial cells in this model.

The cornea lacks vasculature under normal physiologic conditions. Corneal avascularity is maintained by the balance between angiogenic factors including VEGF and bFGF and anti-angiogenic factors including TSP-1 and soluble VEGF receptor I [1,2]. Alkali injury augmented intraocular mRNA expression of *bFGF* and *TSP-1* in CCL3-KO mice to an extent similar to that in WT mice. On the contrary, alkali-induced, enhanced VEGF expression was markedly attenuated in CCL3-KO mice. Because soluble VEGF receptor I, a decoy receptor for VEGF, is constitutively present and acts as a major anti-angiogenic factor in the cornea [1], the reduced VEGF expression in CCL3-KO mice may account for attenuated CNV after alkali injury in these mice. Moreover, CCL3 can augment VEGF production by macrophages. Thus, it is likely that CCL3 induced CNV indirectly by inducing the infiltration and activation of macrophages, a major source of VEGF.

Macrophages are proposed to play crucial roles in tissue repair based on the observations that these cells can abundantly produce various growth and angiogenic factors. As seen with other types of leukocytes, macrophage infiltration is regulated mainly by coordinated actions of adhesion molecules and chemokines. Chemokines bind to their cognate receptors on leukocytes to exert their actions. Macrophages express a limited set of chemokine receptors including CCR1, CCR2, CCR5, and CX3CR1 and exhibit chemotaxis to their ligands [12-14]. CCL3 utilizes two distinct receptors, CCR1 and CCR5 [12], with slight differences in their expression patterns [12,21]. Reduced CNV in CCR5-KO mice prompted us to evaluate the roles of CCL3 and CCR1 in this model. CCL3 deficiency but not CCR1 deficiency reduced alkali-induced CNV. We recently observed that bleomycin-induced intrapulmonary macrophage accumulation and subsequent pulmonary fibrosis was attenuated in CCL3-KO and CCR5-KO mice but not in CCR1 KO mice [34]. This suggests that CCR5 expressing cells are distinct from CCR1 expressing cells. Indeed, a double-color immunofluorescence analysis demonstrated that CCR5 expressing cells did not express CCR1 simultaneously (unpublished data). Thus, CCL3 may generally regulate macrophage functions by binding CCR5 expressed on their surface but not CCR1.

A partial reduction of macrophage infiltration by CCL3 deficiency suggests a contribution of other chemokines such as CCL2 and CX3CL1 to macrophage infiltration. This may further indicate heterogeneity of monocytes/macrophages in terms of chemokine receptor expression patterns as previously suggested by Geissmann and colleagues [35] who proposed the presence of two blood monocytes consisting of inflammatory CX3CR1^low^CCR2^+^ and resting CX3CR1^high^CCR2^-^ populations. Macrophages are presumed to exert pro-angiogenic actions under various situations [24-28], but Apte and colleagues [17] demonstrated anti-angiogenic activities of macrophages in CNV. These observations suggest a functional heterogeneity among macrophages during the angiogenetic process. Indeed, we demonstrated that CX3CR1 positive macrophages could dampen alkali-induced CNV by producing anti-angiogenic molecules [18], which is in contrast to the observations on CCR2-deficient mice [15]. We previously revealed that the CCL2/CCR2 interactions were involved in VEGF production [18] and observed that CCR5 expressing cells simultaneously expressed CCR2. Thus, CCR5 deletion reduced the number of CCR2 expressing macrophages, the cells that can express VEGF, and as a result, this reduction eventually prevented alkali-induced CNV.

Several independent groups have reported the presence of resident macrophages, dendritic cells, langerhans cells, and T cells in normal corneas [36-39]. The number of resident macrophages in the normal corneal stroma is around 100 per mm^2^ [36], and in the current experimental conditions, we detected few, if any, F4/80 positive or CD68 positive macrophages in the normal corneas [18]. Thus, it is not likely that corneal resident macrophages contribute directly to CNV.

However, the simple dichotomy of monocytes/macrophages proposed by Geissmann was complicated by the observation that CCR2^-^ and CCR2^+^ monocytes depended on CCR5 and CX3CR1, respectively, when they entered into atherosclerotic plaques [40]. Ambati and colleagues [15] reported that CCR2 deficiency inhibited CNV, but they did not examine the roles of macrophages in this process. Thus, the effects of the CCR2 axis on macrophage infiltration in CNV or the interaction between the CCR2 and CCR5 axis remain unclear. More detailed analysis on this point will clarify the molecular and cellular mechanisms underlying macrophage infiltration and subsequent CNV development.
